# Machine Learning to Calculate Heparin Dose in COVID-19 Patients with Active Cancer

**DOI:** 10.3390/jcm11010219

**Published:** 2021-12-31

**Authors:** Egidio Imbalzano, Luana Orlando, Angela Sciacqua, Giuseppe Nato, Francesco Dentali, Veronica Nassisi, Vincenzo Russo, Giuseppe Camporese, Gianluca Bagnato, Arrigo F. G. Cicero, Giuseppe Dattilo, Marco Vatrano, Antonio Giovanni Versace, Giovanni Squadrito, Pierpaolo Di Micco

**Affiliations:** 1 Department of Clinical and Experimental Medicine, University of Messina, 98122 Messina, Italy; egidio.imbalzano@unime.it (E.I.); luana_orlando@libero.it (L.O.); veronica.nassisi@gmail.com (V.N.); gianluca.bagnato@unime.it (G.B.); giuseppe.dattlo@unime.it (G.D.); agversace@unime.it (A.G.V.); giovanni.squadrito@unime.it (G.S.); 2 Department of Medical and Surgical Sciences, University Magna Græcia of Catanzaro, 88100 Catanzaro, Italy; sciacqua@unicz.it; 3 Department of Electrical, Computer and Biomedical Engineering, University of Pavia, 27100 Pavia, Italy; giuseppe.nato@icloud.com; 4 Department of Medicine and Surgery, Insubria University, 21100 Varese, Italy; francesco.dentali@asst-settelaghi.it; 5 Department of Medical Translational Sciences, Division of Cardiology, Monaldi Hospital, University of Campania “Luigi Vanvitelli”, 80100 Naples, Italy; v.p.russo@libero.it; 6 Unit of Angiology, Department of Cardiac, Thoracic and Vascular Sciences, Padua University Hospital, 35100 Padua, Italy; giuseppe.camporese@aopd.veneto.it; 7 IRCCS Policlinico S. Orsola—Malpighi, Hypertension and Cardiovascular Risk Research Center, DIMEC, University of Bologna, 40126 Bologna, Italy; arrigo.cicero@unibo.it; 8 UTIC and Cardiology, Hospital “Pugliese-Ciaccio” of Catanzaro, 88100 Catanzaro, Italy; marco.vatrano1975@gmail.com; 9 Department of Medicine, BuonconsiglioFatebenefratelli Hospital, 80100 Naples, Italy

**Keywords:** machine-learning, artificial intelligence, SARS-CoV-2, heparin, anticoagulation

## Abstract

To realize a machine learning (ML) model to estimate the dose of low molecular weight heparin to be administered, preventing thromboembolism events in COVID-19 patients with active cancer. **Methods:** We used a dataset comprising 131 patients with active cancer and COVID-19. We considered five ML models: logistic regression, decision tree, random forest, support vector machine and Gaussian naive Bayes. We decided to implement the logistic regression model for our study. A model with 19 variables was analyzed. Data were randomly split into training (70%) and testing (30%) sets. Model performance was assessed by confusion matrix metrics on the testing data for each model as positive predictive value, sensitivity and F1-score. **Results:** We showed that the five selected models outperformed classical statistical methods of predictive validity and logistic regression was the most effective, being able to classify with an accuracy of 81%. The most relevant result was finding a patient-proof where python function was able to obtain the exact dose of low weight molecular heparin to be administered and thereby to prevent the occurrence of VTE. **Conclusions:** The world of machine learning and artificial intelligence is constantly developing. The identification of a specific LMWH dose for preventing VTE in very high-risk populations, such as the COVID-19 and active cancer population, might improve with the use of new training ML-based algorithms. Larger studies are needed to confirm our exploratory results.

## 1. Introduction

COVID-19 is an acute, systemic complex disorder induced by SARS-CoV-2 infection, with heterogeneous manifestations ranging from paucisymptomatic course to life-threatening severe presentation characterized by bilateral interstitial pneumonia and acute respiratory distress syndrome [1]. It has been associated with a hypercoagulable state and thrombotic complications, mainly in its critical form [2]. Although the American College of Chest Physicians guidelines emphasize treatment of an acute pulmonary embolism as soon as possible, using parenteral anticoagulants, such as subcutaneous low-molecular-weight heparin (LMWH) [3], the exact therapeutic dose and side effects monitoring remain uncertain [4]. COVID-19 have severely impacted care services of fragile groups, and in particular cancer patients, with a significant reduction in the intensity and quality of care [5,6,7] and also a reduced life expectancy if infected by SARS-CoV2 [8]. Neoplastic patients have a state of basic hypercoagulability which exposes them to greater risk of deep venous thrombosis (DVT) and pulmonary embolism (PE) [9,10,11], even if not immediately manifested. At the basis of this are numerous components: the triad of Virchow (the alteration of the vessel wall, the hematic stasis, and the hemostasis), quantitative and qualitative alterations of the platelets and the leukocytes, prothrombotic activity of the same tumor cells, compressive tumor mass stasis, onset of infections, and forced bed rest [12]. SARS-CoV2 pneumonia increases mortality in patients with thoracic tumors [13] and in patients with chemotherapy treatment. Robin Park et al. [14] in a meta-analysis of 16 retrospective and prospective studies, with 3558 patients, show an increased mortality in patients under active chemotherapy treatment, compared to not active chemotherapy. For this reason, a correct evaluation of antithrombotic therapy is essential in oncologic patients, and able to reduce mortality, especially when the appropriate dosage of low molecular weight heparin (LMWH) is administered [15,16]. Therefore, we employed an approach based on machine learning (ML), a branch of computer science that can be considered a close relative of artificial intelligence, to achieve, through an algorithm, the correct anticoagulant therapy to be administered in primary prevention to COVID-19 patients with active cancer. There are different mechanisms that allow an intelligent machine to improve its capabilities and performance over time. The machine will be able to learn to perform certain tasks by improving, through experience, its skills, responses and functions. At the basis of machine learning there is a series of different algorithms which, starting from primitive notions, will be able to make a specific decision rather than another or carry out actions learned over time. Machine learning techniques, compared with traditional statistical models, have many advantages including high power and accuracy, the ability to model non-linear effects, the interpretation of large genomic data sets, robustness to parameter assumptions, and the ability to dispense with a normal distribution test [17].

## 2. Patients and Methods

**Figure 1:**
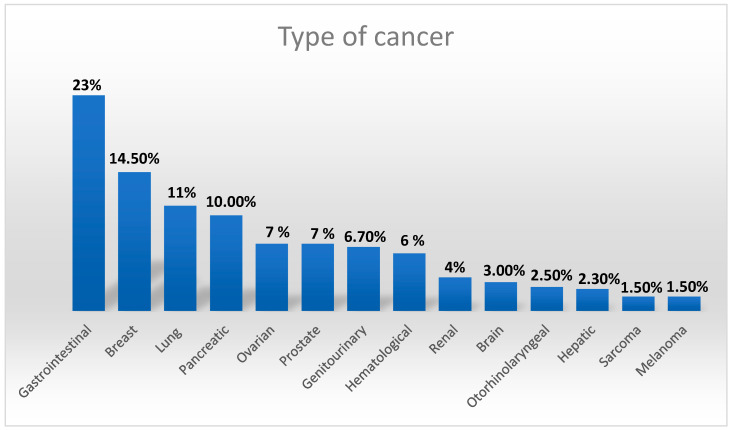
Different types of cancer in 131 enrolled patients.

We included 140 patients with active cancer (defined as diagnosis or treatment in the last 6 months, recurrence or malignant tumor locally advanced or with metastasis, or haematological tumour not in complete remission [18], who were hospitalized in the COVID Hospital of the University Policlinic of Messina, from March 2020 to February 2021. Data were collected from computerized medical charts. The diagnosis of COVID-19 infection was undertaken with a SARS-CoV-2 nasopharyngeal swab by reverse transcription-polymerase chain reaction (RT-PCR). The outcome of interest was the occurrence of a VTE during hospitalization while patients with a known diagnosis of pulmonary embolism or venous thrombosis at admission were excluded. We also excluded from the study patients who did not require low molecular weight heparin prophylaxis or who were already being treated with VKA/DOACs, and patients with a known diagnosis of pulmonary embolism or venous thrombosis. All patients included in the study underwent LMWH at a prophylactic dosage according to the International Guidelines and Medenox Samama trial [19]. The study was approved by the local Ethics Committee and all patients or healthcare decision-makers provided written or oral consent to their participation in the registry. The original dataset with 140 patients and 36 characteristics is presented in Figure S1 and Table S1; nine patients were not considered in the study because they had many missing values, so the final number of patients was reduced to 131. Additionally, we have eliminated redundant and unnecessary features for our study; in this way we obtained the final dataset, used to train the model. In the final dataset we considered 19 variables (see Table S2), all collected at the time of hospitalization, before patients began therapy with LMWH: age, sex, body mass index (BMI), d-dimer levels, platelet count, fibrinogen levels, daily dose of heparin, creatinine, NT-proBNP, mechanical ventilation, fraction of inspired oxygen (FiO_2_), total bilirubin, Glasgow Coma Scale, systolic blood pressure, history of hypertension and/or coronary heart disease, use of ACE inhibitors or angiotensin receptor blockers and thromboembolic events (VTE). Cancer characteristics are shown in Figure 1.

### 2.1. Model Development

In the machine learning approach, the development of the model is divided into three different interconnected phases: target definition, data preparation, and model selection.

The dependent variable y, that is, the target variable, is the “VTE” characteristic, a dichotomous variable that associates the value 1 to patients who have experienced venous thromboembolism and the value 0 to those who did not present this condition. It is a binary classification task, as the machine learning algorithm learns a set of rules, with the aim of distinguishing between two exclusive possible classes: the occurrence and non-occurrence of venous thromboembolism.

Data preparation is one of the most delicate phases of the process, as making a mistake at this stage could compromise the entire work. In this sense, we performed intermediate steps to model and make them usable. It was necessary to manage the missing data as our database had samples with some unspecified values. Assuming that certain fields have been neglected in the detection and considering that most computational tools are not able to handle missing values, as they would produce unpredictable results if we decided to ignore them, it was essential to deal with them before proceeding with the analysis. We then located the missing values as placeholder strings from the Not a Number (NaN) value. Once this was done, the easiest way to manage such data would have been to delete the feature or sample that had such gaps directly from the database. However, we decided not to consider this solution because of the small size of our dataset, as we could delete information useful for the entire process, as well as further reducing its size. One of the most common alternatives is to use interpolation techniques, useful for replacing missing values based on the other samples in the dataset. In our case we chose to use the “mode value” of the relevant column. Finally, before proceeding with the model selection phase, it was necessary to use scaling techniques such as normalization. The goal of normalization is to change the values of the numeric columns in the dataset to use a common scale, without compromising differences between ranges of values or loss of information. We carried out data normalization using the MinMaxScaler class of the scikit-learn pre-processing module (scikit-learn is an important python library in machine learning as it provides a wide range of supervised and unsupervised learning algorithms).

After completing the data preparation steps, we defined the best performing models to be used in our project. We must take into account that each classification algorithm has its inherent flaws and no model can boast absolute superiority; the performance of a classifier, its computational power and its predictive capacity, depend to a large extent on the data that are available for learning. Therefore, it is highly recommended to compare a number of different algorithms, in order to train them and then select the model that offers the best performance.

### 2.2. Limitations of the Study

We worked with a dataset with a limited number of samples and a large amount of characteristics; however, we decided to not reduce further the characteristics considered important for the study, taking into account that we wanted to create a starting model that can be used as a basis for a possible repopulation of the dataset. In this work we compared five of the best known classification models related to supervised learning, with the aim to identifying the best one; these were: logistic regression, decision tree, random forest, support vector machine and Gaussian naive Bayes.

### 2.3. Performance Evaluation

**Figure 2:**
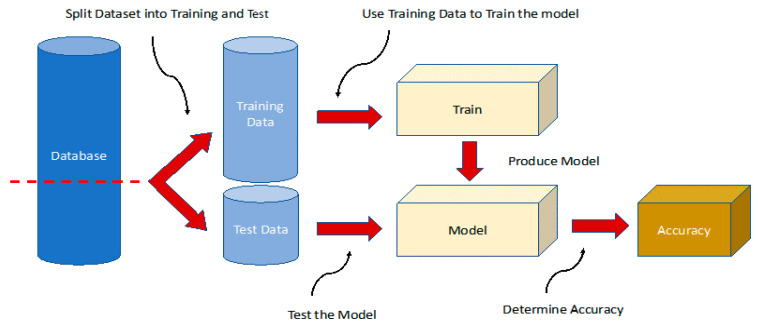
Training and validation scheme for machine learning methods. The database is split, and 70% of the data are used for training and validation of the method and 30% for testing. The model is trained with a training set and scored on the test set (metrics), and then the process is repeated k-times. After this training, pattern discrimination is then tested in a different subset of patients (test set, 30% of the database). The whole process is then repeated until the learning stabilizes and stops improving. The results presented in this study are obtained from the evaluation of this subset.

We divided our dataset into two new sets, which were used respectively as a training set to inform and optimize the machine learning model, and as a test set to evaluate its performance (see Figure 2). This was crucial to test whether the learning algorithm performed well on the training dataset and on any new data. We avoided including resampling techniques such as bootstrapping or cross-validation as they did not bring any benefit in terms of preventing overfitting. This problem was inherent in the nature of the data available to us; the size of the dataset in terms of samples and characteristics considered as well as the non-homogeneity of the reference target made this problem inevitable without increasing the number of samples available.

Using a scikit-learn function, we divided the X, representing the features, and Y the target in a random way, with a ratio of 30% for the test data and 70% for the training data. Next, we created a dictionary of models, containing the name of the classifiers as keys and an instance of the latter as values.

We defined a method, which would take the X and Y matrices of the train and test set as input and apply to them all the classifiers defined in the dictionary.

We created a table containing the accuracy values of the various models and we implemented what could potentially have been the most suitable model for our needs.

We created a matrix to understand whether some features were redundant with each other, evaluating the existence of a possible correlation between the various features, using a function of the seaborne library.

It was also necessary to create a graph showing the correlation coefficients between the characteristics and the target, in order to assess if a characteristic had a greater impact on the desired result. Using a confusion matrix, we could verify the answers provided by the system to establish their reliability.

To prove the validity of our model, we created a particular function in python. We started from a patient that the machine had predicted to have VTE = 1; after that we created a cycle that at each iteration lowered all parameters by 1/20, except the parameters of the binary characteristics that were set before; for example, the sex characteristic was set to 1 and so on. Then we varied the heparin dose starting from a value of 0.1 (10 mg), observing changes in the VTE. The cycle was interrupted when a patient was found in which the VTE characteristic passed from 1 to 0 for a certain dose of heparin.

## 3. Results

**Figure 3:**
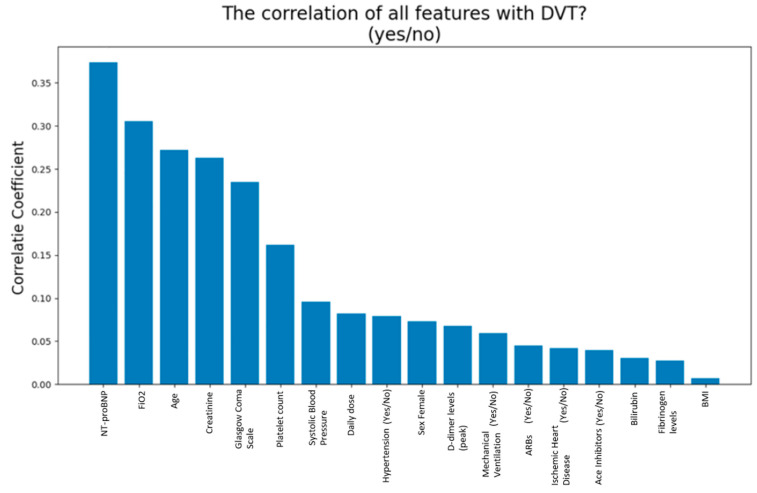
Correlation of all features with VTE. The figure shows the correlation coefficients between all characteristics (*n* = 18) and the VTE characteristic. NTpro-BNP is the variable with the higher degree of correlation with VTE.

We included 131 patients, whose general baseline characteristics are described in detail in Table 1 and Table 2. VTE occurred in 30 patients (23%); among these, 15 patients were female (50%), and 63% had hypertension. The clinical characteristics that have shown statistical significance in patients who developed VTE were: age, creatinine, Glasgow Coma Scale and NT-proBNP. A more detailed description is shown in Table 3 and Table 4. Among all the characteristics showing a greater impact on the model, ’NT-proBNP’ was the most relevant. (Figure 3).

The performance of the ML methods, working with 19 variables (“reduced model”) in the subgroup of patients randomly selected for testing and validation, is shown in Table 5. A good first evaluation metric is the accuracy of the test score. In this sense, we can observe how the accuracy of these methods varied from 67% to 81%. Specifically, we obtained a test score value equal to 81.39% with logistic regression, 79.06% with naive Bayes, 76.74% with random forest, 72.09% with linear SVM”, and 67.44% with decision tree. The “logistic regression” appeared therefore the most efficient; this algorithm was able to predict all events almost without errors.

**Figure 4:**
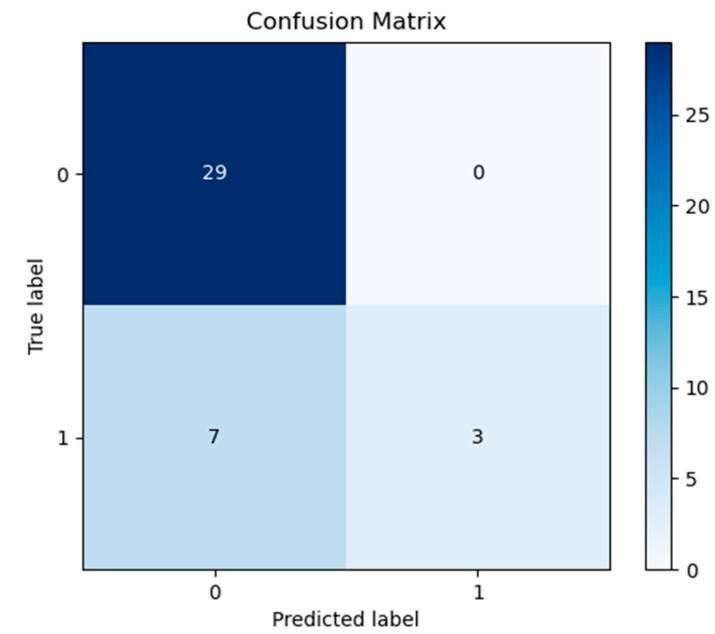
Confusion Matrix. On the main diagonal the predictions made by the machine are reported. Thus, the model was able to correctly answer 29 times in order to identify the true negative group and three times in order to identify the true positive, while it made an error seven times for the false negative. No false positive was detected.

Once the best performing model was chosen, this was implemented separately. Equations used to measure the performance is shown in Figure S2, while in Table S3 we reported the values obtained from the responses evaluated in terms ofpositive predictive value, sensitivity and F1-Score. For the answer 0, we obtained as values of that metric respectively: 81%, 100%, 89%, having a support as 29 sample; for the answer 1, instead we obtained values of: 100%, 30%, 46%, in this case considering a support as 10 samples. We have created a confusion matrix (Figure 4) to make the answers obtained clearer. On the main diagonal the predictions correctly made by the machine are reported, so this was able to answer correctly “0” 29 times and “1” three times, while it made an error seven times by answering “0” when the correct answer was “1”. The ratio of the sum of the elements of the diagonal to all the elements of the confusion matrix is called “Accuracy”. However, we believe it is appropriate to specify that sometimes accuracy can be misleading, especially in scenarios such as ours in which there is a large class imbalance. A model can predict the value of the majority class for all predictions and achieve a high classification accuracy. However, this model is not useful. Additional measures to Accuracy are required to evaluate a classifier, for this reason we included positive predictive value, sensitivity and F1-Score.

Using the function described in the previous paragraph, we could provide the case of a patient as proof of concept, for which the machine returns the exact dose of heparin to be administered, so that it does not manifest venous thromboembolism. The patient characteristics are reported in Table 6. About this patient, the machine predicts the development of venous thrombosis with a dose of heparin <99 mg (VTE = 1), while he does not develop this pathology for a dose ≥99 mg (VTE = 0).

The reduced number of samples in the dataset used represents the only limit of the machine learning training phase.

## 4. Discussion

To date, the application of artificial intelligence has allowed satisfactory results to be achieved in the world of medicine, and a growing body of data is emerging [20,21,22,23], including COVID-19 research. [24,25]. Indeed, in our study we aimed at exploring the application of ML in predicting the appropriate dose of LMWH in a specific fragile population with COVID-19 in order to assess the risk of VTE development.

Of note, according to Samama et al., prophylactic treatment with 40 mg per day of enoxaparin subcutaneously safely reduces the risk of venous thromboembolism in patients with acute medical illnesses [26]. Despite being an acute disease, COVID-19 seems to require a different therapeutic approach. In our study population, treated with the prophylactic dosage of LMWH, as suggested by Samama et al., 23% developed VTE. The question is: are there any specific predictive factors or laboratory parameters of high thromboembolic risk in patients with COVID-19? From a pathophysiological point of view, the prothrombotic state observed in COVID-19 seems to start from the dysfunction of endothelial cells induced by infection, resulting in an excess of thrombin generation and fibrinolysis shutdown; furthermore, the hypoxia found in severe COVID-19 can further stimulate thrombosis through not only increasing blood viscosity, but also a hypoxia-inducible transcription factor-dependent signaling pathway. For this reason, occlusion and micro thrombosis formation in pulmonary small vessels of critical patients with COVID-19 has been reported in several cases, according to a recent lung organ dissection study [27]. Furthermore, the correlation between hypercoagulability status and active cancer has long been documented [28], varying according to the types of cancer. In particular, it has been demonstrated that patients with cancer of mesothelium/soft tissues are more likely to develop thromboembolic events and, in turn, a poor prognosis [29]. This can be, at least partly, explained by the direct release of prothrombotic molecules by the tumor cells and also by an aberrant activation of the coagulation cascade by endothelial and platelet cells [30].

Indeed, one of the first reported features of COVID-19 was its association between the hypercoagulable state (elevated D-dimer levels, fibrin degradation products, and prolonged PT and aPTT) and mortality [31].

In our study NT-proBNP has taken on an important aspect, which is significantly high in patients who have developed VTE compared to those who have not. Many data in the literature confirm our results, showing how patients with severe COVID-19 and heart failure had not only higher levels of cardiac biomarkers, as one might expect, but also a poorer prognosis, worse outcome and higher mortality [32,33,34]. As a sign of myocardial stress, NT-proBNP increase could be due to a cytokine storm in response to the infection trigger and to the direct action of the virus on the heart walls [35,36]. More accurately, NT-proBNP appears as the best representative prognosis biomarker in COVID-19 disease [37].

Due to the observation of high incidence of VTE in our COVID-19 study population, treated with standard dosages of LMWH, we tried to create a system capable of providing a tool to obtain the dose of LMWH to be administered in patients affected by COVID-19 considering their high risk of thromboembolic events.

Once the dataset was arranged, it was divided into two sections, using one part of the data to carry out the machine training operations, and the other part to carry out tests, to query the machine on unknown data and, therefore, to obtain the benefits of the latter.

Tests have been carried out to verify which of the various machine learning algorithms offered the best performance. In this sense, the logistic regression algorithm has been identified as the best performing. Focusing on the implementation of the latter, we carried out targeted tests, interrogating the machine with patient data not used in training, in order to understand its behavior. The results obtained showed how our system succeeds in its intent: in one patient the machine predicts venous thrombosis with a dose of heparin <99 mg (VTE = 1), while this condition does not occur for a dose ≥99 mg (VTE = 0). The possibility of predicting the correct dose of anticoagulant treatment in a patient at high-risk of VTE would allow the therapeutic strategy to be optimized in the shortest time possible and to ensure a better quality of life, possibly reducing one of the most frequent causes of death in this class of patient.

These results may provide a prediction regarding the dose of heparin to be administered in frail patients at high risk of developing VTE due to active cancer and with ongoing COVID-19.

The identification of the minimum effective dose of LMWH for a patient with COVID-19 and active cancer could improve with similar analyses with larger datasets, as required also by the machine learning itself; working with a larger number of samples, in fact, may reduce the recorded overfitting level.

## 5. Conclusions

The world of machine learning and, even more generally, of artificial intelligence is constantly developing. The continuous growth of demand means that new techniques are being developed or refined. In this sense, we aim to refine our system, using new training algorithms in order to observe if their performance might improve the outcome in very high-risk patients, as represented by subjects with concurrent COVID-19 and active cancer, two clinical diseases associated per se with an increased rate of VTE and fatal VTE. These preliminary results might prove useful as the first step towards possible future developments. A larger dataset may be useful for confirming our results and improving current knowledge in order to refine the model.

## Figures and Tables

**Table 1:** Characteristics of COVID-19 study population. BMI = Body Mass Index; FiO_2_ = fraction of inspired oxygen; GCS. = Glasgow Coma Scale; SBP = Systolic Blood Pressure.

All Patients *n* = 131	Mean	SD	Min	Max
Age (years)	71	15	18	100
BMI (kg/m^2^)	24.35	3.09	16.53	33.3
D-dimer (ng/mL)	1.89	1.71	0.27	9.3
Platelet count (mm^3^)	251.28	104.51	31	490
Fibrinogen (mg/dL)	494.59	149.88	152	991
Daily dose	0.5	0.29	0.3	3.2
Creatinine (mg/dL)	0.97	0.56	0.3	3.1
FiO_2_ (%)	34.9	17.81	21	80
Bilirubin (mg/dL)	0.58	0.26	0.16	1.31
GCS.	12.91	2.53	3	15
SBP (mmHg)	122.56	16.16	68	160
NT-ProBNP	1541.87	4489.72	17	33,873

**Table 2:** Baseline characteristics of COVID-19 patients. ARBs = Angiotensin Receptors Blockers.

All Patients (*n* = 131)	
Mechanical Ventilation	
Yes	40 (31%)
No	91 (69%)
Hypertension	
Yes	75 (57%)
No	56 (43%)
Coronary Artery Disease	
Yes	15 (11%)
No	116 (89%)
Ace Inhibitors	
Yes	21 (16%)
No	110 (84%)
Arbs	
Yes	37 (29%)
No	94 (71%)
Sex Female	
Yes	65 (49%)
No	66 (51%)

**Table 3:** Characteristics of patients who developed VTE and who not. BMI = Body Mass Index; FiO_2_ = fraction of inspired oxygen; GCS = Glasgow Coma Scale; SBP = Systolic Blood Pressure.

All Patients *n* = 131		VTE	(*n* = 30)		Not VTE	(*n* = 101)	
	Mean	Median	DS	Mean	Median	DS	Test *t*
Age (years)	78	82	13.3	68	68	14.9	0.001711
BMI (kg/m^2^)	23.9	23.28	3.58	24.42	24.77	2.98	0.498998
D-dimer (ng/mL)	1.74	1.1	1.31	1.95	1.27	1.82	0.551463
Platelet count (mm^3^)	241.41	240	92.24	252.94	225	108	0.60452
Fibrinogen(mg/dL)	503.4	470	198.08	493	476	133.78	0.745607
LMWH Daily dose	0.5	0.4	0.18	0.47	0.4	0.16	0.353239
Creatinine(mg/dL)	1.24	1	0.81	0.89	0.8	0.43	0.00275
FiO_2_ (%)	38.3	35	17.21	33.8	21	17.99	0.228329
Bilirubin (mg/dL)	0.56	0.53	0.21	0.58	0.54	0.26	0.792944
GCS	11.8	12.5	2.57	13.2	15	2.44	0.007232
SBP (mmHg)	125.3	127.5	20.77	121.66	120	14.59	0.278259
NT-ProBNP(ng/L)	4608.43	876.5	8345.56	581.97	187.5	1131.78	0.00002

**Table 4:** Dichotomous characteristics of COVID-19 patients according to VTE development.

	VTE (*n* = 30)	Not VTE (*n* = 101)
Sex (female)	15 (50%)	48 (47%)
Mechanical ventilation	8 (27%)	32 (32%)
Hypertension	19 (63%)	17 (17%)
Coronary heart disease	4 (13%)	10 (10%)
Ace inhibitors	4 (13%)	17 (17%)
ARBs	10 (33%)	28 (28%)

ARBs = Angiotensin Receptors Blockers.

**Table 5:** Accuracy of five classifiers. The test score values represent the performance of the various models. The model with the highest test score is to be considered the best performing.

	Classifier	Train Score	Test Score	Train Time
1	Logistic Regression	0.862069	0.813953	0.046875
2	Naive Bayes	0.816092	0.790698	0.000000
3	Random Forest	1.000000	0.767442	2.093750
4	Linear SVM	0.793103	0.720930	0.000000
5	Decision Tree	1.000000	0.674419	0.000000

**Table 6:** Characteristics of the patient-proof. BMI = Body Mass Index; FiO_2_ = fraction of inspired oxygen; GCS = Glasgow Coma Scale; ARBS= Angiotensin Receptor Blockers.

Patient Proof Characteristics	
Age (Years)	71
Sex (male/female)	1
BMI (kg/m^2^)	20.16
D-Dimer Levels (peak)	0.42
Platelet Count (mm^3^)	111
Fibrinogen Levels (mg/dL)	298
Daily Dose (mg)	99
Creatinine (mg/dL)	1.7
Mechanical ventilation (yes/no)	1
FiO_2_ (%)	26
Bilirubin (mg/dL)	0.59
Glasgow Coma Scale	11
Systolic blood pressure	135
Hypertension (yes/no)	1
Coronary arterydisease (yes/no)	0
Ace inhibitors (yes/no)	0
ARBs (yes/no)	0
NT-proBNP (ng/L)	24,904

## Data Availability

The datasets used and/or analysed during the current study are available from the corresponding author on reasonable request.

## Supplementary Materials

The following supporting information can be downloaded at: https://www.mdpi.com/article/10.3390/jcm11010219/s1, Figure S1: Final database used in our study consists of 132 samples and 19 characteristics, Figure S2: Equations used to measure the performance, Table S1: List of 36 variables, full model, Table S2: List of 19 variables, reduced model, Table S3: Metrics used to measure the performance.

## Limitation

Further data collection is needed.
