# Supplementary material for: Machine Learning to Calculate Heparin Dose in COVID-19 Patients with Active Cancer
Source: J Clin Med. 2021 Dec 31;11(1):219. doi: 10.3390/jcm11010219 (PMC8746167; doi:10.3390/jcm11010219)
Supplement: Supplementary file 1 [file jcm-11-00219-s001.zip › jcm-1497051-supplementary.pdf]

Age (years)	Sex (male/female)	IMC	D-dimer levels (peak)	Platelet count	Fibrinogen levels	Daily dose	Creatinine (mg/dL)	Mechanical ventilation (yes/no)	FiO2 (%)	Bilirubin (mg/dl)	Glasgow Coma Scale	Systolic blood pressure	Hypertension (yes/no)	Ischemic Heart Disease (yes/no)	ACE inhibitors (yes/no)	ARBs (yes/no)	NT-proBNP	TEV?(yes/no)
90	1	21,64	4,00	207000	460	0,4	0,8	0	21	0,64	13	120	1	0	0	0	150,00	0
75	1	27,34	1,27	443000	381	1,2	0,8	0	21	0,51	15	120	1	0	0	1	1097,00	0
74	1	20,03	1,87	328000	401	0,4	0,4	0	31	0,44	9	115	0	0	0	0	308,00	1
74	0	20,28	1,94	270000	850	0,8	1,2	0	60	0,29	10	130	1	0	1	0	853,00	1
81	0	25,95	1,66	214000	498	0,3	1,7	0	21	0,27	15	115	1	0	0	1	5863,00	0
62	1	24,22	0,36	230000	675	0,6	0,5	0	21	0,24	10	110	1	0	0	0	159,00	0
65	0	16,53	0,38	200000	340	0,8	0,8	0	21	0,26	13	115	0	0	0	0	188,00	0
76	0	25,39	0,27	239000	418	0,4	1	0	21	0,48	15	115	0	0	0	0	4159,00	0
59	0	27,34	0,48	155000	427	0,4	0,8	0	21	0,35	15	130	0	0	0	0	18,00	0
49	0	19,59	0,27	149000	152	0,4	1	0	21	0,94	15	125	0	0	0	0	37,00	0
84	1	27,34	1,17	140000	633	0,4	0,9	0	28	0,35	15	120	0	0	0	0	321,00	0
81	1	22,04	1,01	264000	388	0,6	1,7	0	24	0,48	12	120	1	1	1	0	2102,00	0
39	0	20,98	0,51	178000	594	0,4	0,4	1	24	0,80	15	120	0	0	0	0	29,00	0
91	1	21,48	0,48	141000	351	0,4	2,3	1	28	0,75	15	160	1	0	0	1	33873,00	1
55	0		0,27	130000	574	0,5	0,3	0	21	0,47	13	110	1	0	1	0	43,00	1
61	0		0,67	118000	575	0,4	1	1	21	0,55	15	130	0	0	0	0	159,00	0
79	0		4	106000	632	0,3	2	1	60	0,67	13	120	1	0	0	1	697,00	0
69	0	19,15	1,18	172000	584	0,8	0,5	0	21	0,90	10	145	1	0	1	0	333,00	0
85	1		1,74	272000	691	0,4	0,5	0	35	0,27	11	120	0	0	0	0	1715,00	1
83	0	18,29	0,27	239000	278	0,4	0,7	0	21	0,58	15	120	1	0	0	1	599,00	0
67	1	29,14	0,31	213000	445	0,4	0,8	0	21	0,36	15	100	0	0	0	0	391,00	0
55	0	21,60	0,39	243000	385	0,8	1,1	0	21	0,40	15	120	0	0	0	0	27,00	1
84	1	24,22	0,60	137000	551	0,5	0,7	0	21	0,37	15	135	1	0	0	1	151,00	0
89	0	25,71	0,95	165000	524	0,4	1,5	0	21	0,27	15	130	1	1	1	0	1563,00	0
87	1	27,34	4	302000	246	0,8	3,1	0	21	0,92	6	130	1	0	0	0	4907,00	1
72	1	22,49	4	360000	502	0,4	0,9	0	28	0,59	14	150	0	1	0	0	1160,00	0
18	1	16,65	0,27	154000	327	0,4	0,4	0	21	0,70	15	100	0	0	0	0	59,00	0
58	1	21,40	2,22	303000	586	0,5	0,6	0	24	0,19	6	90	0	0	0	0	346,00	0
75	1		4	225000	327	0,4	0,8	0	21	0,49	15	130	1	0	0	1	566,00	0
50	0	26,12	1,78	400000	709	0,4	0,8	0	35	0,62	15	110	0	0	0	0	34,00	0
55	0	25,71	0,44	170000	577	0,4	0,7	0	21	0,36	15	115	0	0	0	0	17,00	0
74	1		4	109000	450	0,4	1,9	1	40	1,31	3	100	1	0	0	1	1419,00	0
73	1	25,39	0,34	186000	481	0,4	0,6	0	21	0,49	15	150	1	0	1	0	82,00	0
79	0		4	158000	789	0,4	1,5	0	60	0,69	8	145	0	0	0	0	2833,00	1
74	0	21,60	0,62	132000	245	0,4	0,8	0	21	0,25	15	130	1	0	0	1	221,00	0
86	1		0,93	162000	168	0,4	1,5	0	68	0,56	13	140	1	0	0	1	900,00	1
43	1	33,30	4	167000	526	0,4	0,7	0	21	0,21	10	150	1	0	0	1	110,00	0
89	0	23,44	1,24	176000	520	0,4	2	0	21	0,34	12	110	1	0	0	1	5890,00	0
55	0	27,68	0,53	162000	700	0,4	0,9	0	21	0,72	15	115	0	1	0	0	96,00	0
53	1	20,00	0,32	223000	213	0,4	0,3	0	21	0,31	13	140	1	0	0	1	81,00	0
51	0	25,47	0,30	158000	689	0,4	0,9	0	24	0,54	15	115	1	0	1	0	99,00	0
81	1	25,71	3,15	378000	296	0,4	0,5	0	24	0,51	9	140	1	0	0	1	4852,00	1
63	1		1,15	317000	779	0,4	0,4	1	21	0,34	9	110	0	0	0	0	173,00	1
100	1	24,17	1,14	225000	606	0,3	0,9	0	21	0,47	15	130	0	1	0	0	957,00	0
86	0	22,09	1,31	143000	331	0,4	0,7	0	28	0,24	15	100	1	0	0	1	208,00	0
51	1	22,31	0,65	141000	397	0,4	0,5	0	21	0,89	15	90	0	0	0	0	122,00	0
68	1	26,67	0,39	379000	425	0,4	0,6	0	21	0,30	10	115	0	0	0	0	50,00	0
66	1	24,97	0,27	171000	319	0,4	0,4	0	21	0,34	15	110	0	0	0	0	75,00	0
87	1	22,66	0,56	155000	429	0,8	1,9	0	21	0,16	15	115	0	1	0	0	1402,00	0
95	1	23,78	1,05	182000	460	0,4	1,2	0	35	0,54	13	130	0	0	0	0	14228,00	1
39	0	19,53	0,27	170000	420	0,4	0,6	0	21	0,31	12	120	0	0	0	0	40,00	0
58	0		1,66	354000	856	0,4	0,51	0	60	0,80	15	150	1	0	1	0	117,00	0
57	0	22,86	2,27	150000	476	0,4	1,1	0	21	0,76	15	120	0	0	0	0	524,00	0
78	1	20,81	0,72	186000	496	0,3	0,5	0	21	0,36	11	110	0	0	0	0	213,00	1
91	0	17,72	0,82	133000	715	0,4	1,5	0	21	0,78	15	120	0	0	0	0	2779,00	1
76	1	21,48	0,35	225000	380	0,4	0,7	0	21	0,24	15	140	1	0	0	1	439,00	0
67	0		2,56	153000	527	0,4	0,8	0	21	0,38	15	130	0	0	0	0	199,00	0
37	0	25,71	1,51	212000	425	0,4	0,9	0	21	0,29	15	130	0	0	0	0	33,00	0
89	1		1,45	172000	380	0,8	1,2	0	28	0,74	12	155	1	1	1	0	301,00	1
87	1		4	191000	405	0,4	1,9	0	21	0,30	9	68	0	0	0	0	14600,00	1
65	0		4	405000	991	0,8	0,9	1	60	0,72	10	110	1	1	0	1	571,00	1
61	1	22,89	0,70	183000	595	0,4	1,1	0	21	0,26	15	130	1	0	1	0	75,00	0
86	0	22,22	0,77	225000	379	0,4	0,8	0	32	0,75	15	100	0	0	0	0	642,00	0
83	1	31,25	0,67	163000	584	0,5	0,7	0	50	0,50	15	100	1	0	0	0	389,00	1
82	0		4	119000	380	0,4	1,4	0	31	1,25	15	120	1	0	1	0	192,00	0
71	1	22,49	1,76	356000	569	0,4	0,7	0	24	0,58	15	140	1	0	0	1	81,00	0
87	1	25,10	0,59	267000	371	0,4	1,1	0	21	0,35	15	110	1	1	1	0	469,00	0
62	0	27,68	0,85	235000	619	0,4	3,1	0	21	0,24	15	110	0	1	0	0	328,00	0
73	0		0,78	202000	470	0,4	2,9	0	21	0,35	15	130	1	1	0	1	4135,00	1
85	0	20,48	0,46	182000	539	0,4	2,6	0	21	0,45	15	115	1	0	0	1	403,00	1
52	0	22,22	0,57	180000	643	0,4	0,6	0	21	0,64	15	110	1	0	0	0	118,00	0
42	1		2,11	190000	553	0,5	0,6	0	21	1,03	12	130	0	0	0	0	36,00	0
57	0	25,71	0,29	194000	419	0,4	0,8	0	21	0,55	15	110	1	0	1	0	32,00	0
57	1		0,27	249000	262	0,4	0,4	0	21	0,52	15	125	0	0	0	0	57,00	0
89	1		4	240000	400	0,4	1	0	60	0,26	14	150	1	0	0	1	5027,00	1
92	1	26,57	4,00	355000	659	0,8	1,4	0	60	0,66	12	150	1	0	0	1	5803,00	1
54	1	29,38	0,70	280000	570	0,4	0,5	0	21	0,51	15	140	0	0	0	0	31,00	0
88	1	21,08	0,74	181000	441	0,4	0,4	0	21	0,31	15	135	0	0	0	0	220,00	0
81	1		1,63	171000	503	0,4	0,7	0	21	0,58	15	130	0	0	0	0	151,00	0
83	0	23,23	1,22	247000	631	0,8	1,6	0	21	0,56	15	140	1	0	0	0	5208,00	0
70	1	24,44	0,46	177000	435	0,4	0,6	0	21	0,38	15	140	1	0	0	1	66,00	0
90	0	22,20	1,73	87000	527	0,3	2,8	0	40	0,51	8	85	1	0	0	0	27660,00	1
50	0	20,83	0,84	191000	596	0,4	1	0	21	0,54	15	105	0	0	0	0	50,00	0
61	0	25,95	1,92	345000	636	0,4	1,4	0	21	0,26	15	130	1	0	0	1	19,00	0
86	1	22,52	2,57	184000	431	0,4	1	0	21	0,37	15	140	1	0	0	1	1787,00	0
90	1	24,17	0,68	166000	342	0,8	1	1	35	0,24	13	130	1	1	0	1	506,00	1
92	1	23,14	0,69	114000	438	0,4	1,2	0	21	0,24	15	150	1	0	1	0	996,00	0
55	0	23,24	0,99	299000	499	0,4	0,4	0	21	0,80	15	125	1	0	1	0	191,00	1
89	1	20,58	1,39	211000	515	0,4	0,8	0	21	0,53	15	120	1	0	0	1	1496,00	0
76	0	23,88	2,97	31000	427	0,5	0,8	0	21	0,46	15	115	0	0	0	0	187,00	0
70	0	23,44	1,74	230000	415	0,4	0,8	0	24	0,23	12	105	0	0	0	0	114,00	0
76	0	25,71	2,05	121000	400	0,4	1,1	0	21	0,37	15	150	1	0	0	1	88,00	0
49	0	23,88	0,87	161000	623	0,5	1,4	0	28	0,31	15	115	1	0	0	1	32,00	0
90	1	21,40	4	377000	394	0,4	0,8	0	21	0,49	15	125	1	1	0	0	296,00	0
84	1	27,84	2,10	260	390	0,4	0,7	1	52	0,80	12	110	1	0	1	0		0
85	1	25,15	1,80	380	458	0,4	0,8	1	47	1,10	13	130	0	0	0	0		0
84	1	28,12	1,10	410	460	0,4	0,5	1	48	0,60	14	110	0	0	0	0		0
64	0	25,95	4,40	378	380	0,4	1,1	1	44	0,50	14	140	0	0	0	0		0
85	1	30,04	3,90		750	0,8		1	64	1,00	9	150	1	0	1	0		0
63	0	22,98	2,50	430	630	0,4	0,8	1	66	0,70	10	160	0	0	0	0		1
64	0	25,26				0,4		1	70	0,80	10	120	0	0	0	0		0
57	1	26,56	3,90	390	385	0,8	0,6	1	60	1,10	10	140	1	0	0	1		0
59	0	22,20	1,50	340	360	0,4	0,9	1	55	1,00	11	133	0	0	0	0		0
51	0	29,09	1,90	262	370	0,4	1	1	60	0,60	11	137	1	0	0	0	196,00	1
74	0	25,85	5,60	397	791	0,4	0,8	1	68	0,50	11	150	1	0	1	0	218,00	0
83	0	25,14	0,80		330	0,4		1	43	0,60	14	120	0	0	0	0		0
63	1	25,28				0,4		1	46	0,90	13	120	1	0	1	0		0
63	0	25,10	1,40	384	679	0,4	1,2	1	50	0,80	12	135	0	0	0	0	353,00	0
58	1	23,31	0,90			0,4		1	59	0,90	13	120	0	0	0	0		1
53	0	23,15	3,50	366	503	0,8	0,7	1	0,8	0,80	9	110	0	0	0	0		0
55	1	27,55	6,00	412	550	0,8	1,1		0,74	1,20	10	100	0	0	0	0	310,00	0
60	0	22,64	4,50		382	0,4	0,8	1	0,77		9	110	1	0	0	0		0
55	0	23,15	9,30	375		0,4	0,5	1	0,6	0,90	10	110	0	0	0	0	1200,00	0
62	1	27,89	6,50	420	700	0,4	0,7	1	0,63	1,10	9	100	0	0	0	0	320,00	0
66	0	24,77	0,60	170	381	0,4	0,8	0	0,33	0,60	14	115	1	0	0	1	70,00	0
83	0	23,94	0,70	289	370	0,4	1,3	0	0,35	0,90	15	120	1	1	1	0	180,00	0
49	0	23,94	1,40	305	512	0,8	0,4	1	0,7		10	110	1	0	0	0	54,00	0
66	1	25,95		340	412	0,4	0,5	1	0,33	0,40	14	120	1	0	0	1	544,00	0
68	1	27,89	2,50	370	430	0,4	0.9	0	0,35	0,70	15	130	1	0	1	0	60,00	0
73	1	30,43	4,80	432	432	0,8	0,5	1	0,6	0,70	11	120	0	0	0	0	456,00	0
71	0	24,77	5,50	390	550	0,8	0,7	1	0,65	0,90	11	110	1	0	0	1	380,00	0
74	1	26,57	1,10	445	635	0,8	0,6	1	0,63	0,60	10	115	1	0	0	1	885,00	0
66	1	27,55	4,40	480	750	0,8	0,8	1	0,65	0,80	10	110	1	0	0	1	180,00	0
84	1	29,30	0,70	265	212	0,4	0,5	0	0,35	0,90	13	115	1	0	0	0	1400,00	1
68	1	28,58	3,50	395	650	0,4	0,6	1	0,45	0,70	13	110	1	0	0	0	100,00	0
56	0	26,20	7,20	490	680	0,8	1	1	0,63	0,70	9	98	1	0	0	1	270,00	0
73	0	27,78	4,60	460	590	0,4	1,1	1	0,65	0,80	9	110	1	0	0	0	320,00	0
69	0	24,77	2,50	420	512	0,4	0,8	1	0,55	0,80	12	120	0	0	0	0	140,00	0
50	0	23,77	2,30	340	560	0,8	0,7	1	0,66	0,50	11	120	1	0	0	0	40,00	0
77	0	26,73	0,90	380	390	0,8	0,9	1	0,45	0,90	13	130	1	0	0	1	150,00	1
77	0	26,73	0,90	380	390	3.2	0,9	1	0,45	0,90	13	130	1	0	0	1	150,00	0

Supplementary Figure S1. Final database used in our study consists of 132 samples and 19 characteristics. The patients considered were hospitalized into Covid Hospital of University Policlinic of Messina from March to September 2020.

Supplementary Table S1. List of 36 variables, full model. DOB = Date of birth, BMI = Body Mass Index, DVT = Deep Vein Thrombosis, CUS = Compression Ultrasonography, PE = Pulmonary Embolism, ARBs = Angiotensin Receptors Blockers, HF = Heart Failure.

**1.**	Patient's number-ID
**2**	DOB
**3**	Age (years)
**4**	Sex (male/female)
**5**	Date admission in hospital
**6**	In the ICU (yes/no)
**7**	Date diagnosis COVID
**8**	Weight (Kg)
**9**	Altezza (cm)
**10**	BMI
**11**	D-dimer Levels (peak)
**12**	Platelet count
**13**	Fibrinogen levels
**14**	PT (in seconds)
**15**	INR
**16**	DVT signs (yes/no)
**17**	Bilateral CUS (yes/no)
**18**	DVT? (yes/no)
**19**	DVT Territory or PE
**20**	CT scan
**21**	Drug for thromboprophylaxis
**22**	Daily dose (mg)
**23**	Creatinine (mg/dL)
**24**	Mechanical ventilation (yes/no)
**25**	Pa02 (mmHg)
**26**	FiO2 (%)
**27**	Bilirubin (mg/dl)
**28**	Glasgow Coma Scale
**29**	Systolic blood pressure
**30**	Hypertension (yes/no)
**31**	Ischemic heart disease (yes/no)
**32**	Ace inhibitors (yes/no)
**33**	ARBs (yes/no)
**34**	HF (yes/no)
**35**	NYHA (class)
**36**	NT-proBNP

Supplementary Table S2. List of 19 variables, reduced model. ARBs = Angiotensin Receptor Blockers, VTE = Venous Thromboembolism.

**1.**	Age (years)
**2**	Sex (male/female)
**3**	BMI
**4**	D-dimer Levels (peak)
**5**	Platelet count
**6**	Fibrinogen levels
**7**	Daily dose (mg)
**8**	Creatinine (mg/dL)
**9**	Mechanical ventilation (yes/no)
**10**	FiO2 (%)
**11**	Bilirubin (mg/dl)
**12**	Glasgow Coma Scale
**13**	Systolic blood pressure
**14**	Hypertension (yes/no)
**15**	Ischemic heart disease (yes/no)
**16**	Ace inhibitors (yes/no)
**17**	ARBs (yes/no)
**18**	NT-proBNP
**19**	VTE (yes/no)

Precision/Sensitivity: $Se = \frac{Tp}{Tp+Fp}$

Recall: $Re = \frac{Tp}{Tp+Fn}$

F1-score: $F1score = 2 \times \frac{Recall \times Precision}{Recall+Precision}$

Accuracy: $Accuracy = \frac{Tp+Tn}{Tp+Tn+Fp+Fn}$

Supplementary Figure S2. Equations used to measure the performance. Tp is the number of true positive samples, Tn the number of true negative samples, Fn the number of false negative samples and Fp is the number of false positive samples.

Supplementary Table S3. Metrics used to measure the performance. Metrics to measure performance, three types of metrics were used: Precision, Recall and F1-Score.

	Precision	Recall	F1-score	Support
0	0.81	1.00	0.89	29
1	1.00	0.30	0.46	10
Accuracy			0.32	39
Macro avg	0.90	0.65	0.68	39
Weighted avg	0.86	0.82	0.79	39
